# Gamma-oryzanol reduces renal inflammation and oxidative stress by modulating AGEs/RAGE axis in animals submitted to high sugar-fat diet

**DOI:** 10.1590/2175-8239-JBN-2021-0002

**Published:** 2021-06-18

**Authors:** Fabiane Valentini Francisqueti-Ferron, Artur Junio Togneri Ferron, Alessandra Altomare, Jéssica Leite Garcia, Fernando Moreto, Ana Lúcia A. Ferreira, Igor Otávio Minatel, Giancarlo Aldini, Camila Renata Corrêa

**Affiliations:** 1 Universidade Estadual Paulista (UNESP), Faculdade de Medicina, Botucatu, SP, Brasil.; 2 Università degli Studi di Milano, Department of Pharmaceutical Sciences (DISFARM), Milan, Italy.; 3 Universidade Estadual Paulista (UNESP), Instituto de Biociências, Botucatu, SP, Brasil.

**Keywords:** AGE receptor, Inflammation, Antioxidants, Kidney, Obesity, Receptor RAGE, Inflamação, Antioxidante, Rim, Obesidade

## Abstract

**Introduction:**

The receptor for AGEs (RAGE) is a multiligand member of the immunoglobulin superfamily of cell surface receptors expressed in many organs, among them, the kidneys. When activated, RAGE leads to a sequence of signaling that results in inflammation and oxidative stress, both involved in kidney disease pathogenesis. Gamma-oryzanol (γOz) comprises a mixture of ferulic acid (FA) esters and phytosterols (sterols and triterpene alcohols) mainly found in rice, with antioxidant and anti-inflammatory activities.

**Aim:**

To evaluate the effect of γOz to reduce renal inflammation and oxidative stress by modulating AGEs/RAGE axis in animals submitted to a high sugar-fat diet.

**Methods:**

Male Wistar rats (±187g) were randomly divided into two experimental groups: control (n = 7 animals) and high sugar-fat diet (HSF, n = 14 animals) for 20 weeks. After this period, when the presence of renal disease risk factors was detected in the HSF group (insulin resistance, dyslipidemia, increased systolic blood pressure and obesity), the HSF animals were divided to begin the treatment with γOz or continue receiving only HSF for 10 more weeks.

**Results:**

No effect of γOz on obesity and metabolic parameters was observed. However, kidney inflammation and oxidative stress decreased as soon as RAGE levels were reduced in HSF + γOz.

**Conclusion:**

It is possible to conclude that the gamma- oryzanol was effective in reducing inflammation and oxidative stress in the kidney by modulating the AGEs/RAGE axis.

## Resumo

**Introdução:**

O receptor para AGEs (RAGE) é um membro multiligante da superfamília das imunoglobulinas dos receptores de superfície celular expresso em muitos órgãos, entre eles, os rins. Quando ativado, o RAGE leva a uma sequência de sinalização que resulta em inflamação e estresse oxidativo, ambos envolvidos na patogênese de doenças renais. O gama-orizanol (γOz) compreende uma mistura de ésteres de ácido ferúlico (AF) e fitoesteróis (esteróis e álcoois triterpenos) encontrados principalmente no arroz, com atividades antioxidantes e anti-inflamatórias.

**Objetivo:**

Avaliar o efeito do γOz para reduzir a inflamação renal e o estresse oxidativo pela modulação do eixo RAGE/AGEs em animais submetidos a uma dieta rica em gordura e açúcar.

**Métodos:**

Ratos Wistar machos (±187g) foram divididos aleatoriamente em dois grupos experimentais: controle (n = 7 animais) e dieta rica em gordura e açúcar (HSF, do inglês high sugar-fat diet, n = 14 animais) por 20 semanas. Após este período, quando foi detectada a presença de fatores de risco de doença renal no grupo HSF (resistência à insulina, dislipidemia, aumento da pressão arterial sistólica e obesidade), os animais HSF foram divididos para iniciar o tratamento com γOz ou continuar recebendo apenas HSF por mais 10 semanas.

**Resultados:**

Não foi observado nenhum efeito do γOz na obesidade e nos parâmetros metabólicos. No entanto, a inflamação e o estresse oxidativo renais diminuíram assim que os níveis de RAGE foram reduzidos em HSF + γOz.

**Conclusão:**

É possível concluir que o gama- orizanol foi eficaz em reduzir a inflamação e o estresse oxidativo no rim pela modulação do eixo RAGE/AGEs.

## INTRODUCTION

Protein glycation is a complex series of sequential reactions collectively called the Maillard reaction that result in advanced glycation end products (AGEs) formation. Endogenous sources of AGEs can be found in all tissues and ﬂuids where glucose concentration is enough to react with proteins, such as in conditions of hyperglycaemia and diabetes^1^. Moreover, the degradation of glycated proteins, glycolytic intermediates, and degradation of aldoses and ketoses result in the formation of reactive carbonyl species such as glyoxal (G), methylglyoxal (MG), and 3-deoxyglucosone, which are also able to react with proteins to form more AGEs directly^2^. AGEs are also present in ingested food, characterizing exogenous AGEs sources. The content depends on the nutrient composition and how the food is processed (for example, high levels of AGEs are found in roasted, smoked, and baked foods)^1^^,^ 3.

At the cellular level, the damaging effects of AGEs have been attributed to several AGE-binding proteins, such as RAGE (receptor for AGEs), AGEs receptor (AGER) 1, R2, R3, and scavenger receptors such as CD-3647 and SCR-II 4. It is important to emphasize that these cell surface receptors can bind to advanced glycation end products, and also can interact with multiple ligands called multi-ligand receptor, among them: high-mobility group protein (B)1 (HMGB1), S-100 calcium-binding protein, amyloid-β-protein, Mac-1, and phosphatidylserine^5^. Among AGEs receptors, the RAGE is the most notable one, and it triggers oxidative stress and inflammation in both acute and chronic diseases. Specifically, the binding of RAGE leads to a sequence of signaling with the activation of the transcription factor nuclear factor kappa-B (NFκB) resulting in proinflammatory cytokines production, among them tumoral necrosis factor alpha (TNF-α), interleukin-6 (IL-6), and monocyte chemoattractant protein-1 (MCP-1)^6^. 

RAGE activation leads to oxidative stress by inducing nicotinamide adenine dinucleotide phosphate (NADPH)-oxidase (NOX), especially NOX-4. Oxidation driven by the AGEs/RAGE axis induces protein and lipid oxidation leading to the formation of protein carbonyls and lipid-peroxidation, being responsible for lipid-derived reactive carbonyl species which in turn form protein carbonyl adducts (ALEs) which are also RAGE binders, thus sustaining the RAGE activation^7^. Thus, according to the information above described, it is possible to note that the AGEs/RAGE axis is an interface between oxidative stress and inflammation, which are pillars for the development of several diseases, especially in organs that express these receptors for AGEs, as brain, heart, and kidneys^8^. 

The podocyte is the main RAGE expressing cell in the renal glomerulus^1^^,^ 9. It has been described in the literature that RAGE-dependent signal transduction in podocytes leads to apoptosis, production of monocyte chemoattractant protein-1, inflammatory mediators, and oxidative stress via NOX-4, causing renal structural changes, resulting in increased proteinuria and reduced glomerular filtration rate (GFR)^9^. Once GFR is the main way to excrete AGEs, the exacerbation of kidney injury contributes to accumulating AGEs, characterizing a vicious positive feedback of AGEs accumulation through AGEs/RAGE-induced oxidative stress in the course of CKD progression^1^.

In this way, the search for strategies to lower the ligand burden (AGEs and other RAGE ligands) and strategies to dampen RAGE activation have received attention, such as the use of natural compounds as a promising pool of substances to treat diseases^10^^,^ 11. Gamma-oryzanol (γOz) comprises a mixture of ferulic acid (FA) esters and phytosterols (sterols and triterpene alcohols) mainly found in rice, a very important grain in the human diet. A great variety of biological effects have been attributed to γOz, such as antidiabetic, antioxidant, anti-inflammatory, and anti-obesity effects^12^. Other studies have already demonstrated a positive effect of γOz to prevent cardiorenal metabolic syndrome^13^, improve renal disease^10^, and increase muscle growth and sports performance^14^.

Thus, since the AGEs/RAGE ligation is able to induce kidney inflammation and oxidative stress and given the lack of studies that evaluate the effect of γOz in renal AGEs/RAGE modulation, the aim of this study was to evaluate the effect of γOz in reducing renal inflammation and oxidative stress by modulating AGEs/RAGE axis in animals submitted to a high sugar-fat diet. The rationale for using the γOz in this animal model is sustained by the recent literature demonstrating that this class of compounds, besides having a well-established antioxidant activity, exerts a direct antiglycation effect^15^.

## MATERIAL AND METHODS

### EXPERIMENTAL PROTOCOL

All the experiments and procedures were approved by the Animal Ethics Committee of Botucatu Medical School (1150/2015) and were performed in accordance with the National Institute of Health's Guide for the Care and Use of Laboratory Animals. Male Wistar rats (±187 g) were housed in individual cages in an environment-controlled room (22±3 °C; 12 h light-dark cycle, and relative humidity of 60±5 %) and randomly divided into two experimental groups: control (n = 7 animals) and high sugar-fat diet (HSF, n = 14 animals) for 20 weeks. At the 20^th^ week, when the presence of renal disease risk factors was detected in HSF group^10^^,^ 13 (insulin resistance, dyslipidemia, increased systolic blood pressure, and obesity), the animals were divided to begin the treatment with γOzor continue receiving only HSF for 10 more weeks: HSF, n=7 animals and HSF + γOz, n= 7 animals. The HSF diet contained soybean meal, sorghum, soybean peel, dextrin, sucrose, fructose, lard, vitamins, and minerals, plus 25 % sucrose in drinking water; the control diet contained soybean meal, sorghum, soybean peel, dextrin, soy oil, vitamins, and minerals. The nutrients and nutritional composition of each diet was described in our previous study^13^. 

### GAMMA- ORYZANOL

The compound was purchased from Tokyo Chemical Industry Co., Ltd. (Toshima, Kita-ku, Tokyo) (lot.5ZZYLPJ). The γOz used in this study was added in the chow (0.5 w/w) in line with our previous study^13^ in order to simulate the daily consumption of rice of an adult individual in Brazil, according to data from the Family Budget Survey (POF) 2008-2009^16^. 

### NUTRITIONAL PARAMETERS AND OBESITY- RELATED DISORDERS EVALUATION

The nutritional proﬁle considered: final body weight (FBW), adiposity index (AI), insulin resistance, triglycerides levels, and systolic blood pressure (SBP). Body weight was measured weekly. After euthanasia, the fat deposits (visceral (VAT), epididymal (EAT), and retroperitoneal (RAT)) were used to calculate the adiposity index (AI) by the following formula: VAT+EAT+RAT /FBW ×100^17^. 

After 12 h fasting, blood was collected and the plasma was used to measure insulin and biochemical parameters. Blood from fasted animals was collected in tubes containing EDTA and centrifuged at 3500 rpm and the plasma was collected for analysis. Glucose concentration was determined using a glucometer (Accu-Chek Performa, Roche Diagnostics Brazil Limited); triglycerides were measured with an automatic enzymatic analyzer system (Chemistry Analyzer BS-200, Mindray Medical International Limited, Shenzhen, China). The insulin levels were measured using the enzyme-linked immunosorbent assay (ELISA) method using commercial kits (EMD Millipore Corporation, Billerica, MA, USA). The homeostatic model of insulin resistance (HOMA-IR) was used as an insulin resistance index, calculated according to the following formula: HOMA-IR= (fasting glucose (mmol/L) × fasting insulin (µU/mL)) / 22.5^18^. 

Systolic blood pressure (SBP) evaluation was assessed in conscious rats by the non-invasive tail-cuﬀ method with a Narco Bio-Systems^®^ electrosphygmomanometer (International Biomedical, Austin, TX, USA). The animals were kept in a wooden box (50×40 cm) between 38 and 40 °C for 4-5 minutes to stimulate arterial vasodilation^19^. After this procedure, a cuﬀ with a pneumatic pulse sensor was attached to the tail of each animal. The cuﬀ was inﬂated to 200 mmHg pressure and subsequently deﬂated. The blood pressure values were recorded on a Gould RS 3200 polygraph (Gould Instrumental Valley View, Ohio, USA). The average of three pressure readings was recorded for each animal. 

### RAGE LEVELS

Renal tissue (±150 mg) was homogenized (ULTRA-TURRAX^®^ T 25 basic IKA^®^ Werke, Staufen, Germany) in 1.0 mL of phosphate-buffered saline (PBS) pH 7.4 cold solution and centrifuged at 800 g at 4 °C for 10 min. The supernatant (100 µL) was used in analysis. Receptors for advanced glycation end products (RAGE) levels were measured using the enzyme-linked immunosorbent assay (ELISA) method using commercial kits from R&D System, Minneapolis, USA (DY- 1616; 4000- 31.3 pg/mL of detection). The results were corrected according to the protein amount.

### AGES LEVELS

Most AGEs have a characteristic fluorescence. Thus, the determination of AGEs was based on spectrofluorometric detection according to Henle et al.(1991)^20^ and Münch et al. (1997)^21^. Plasma and urine were diluted 1:20 with PBS (phosphate buffer) pH 7.4 and fluorescence intensity was recorded in emission maximum (440 nm) upon excitation at 370 nm (spectrofluorometer Fluoromax-3, Jobin Yvon Horiba, USA). Fluorescence intensity is expressed in arbitrary units (UF/mg protein).

### RENAL INFLAMMATORY PARAMETERS

Inflammation itself is a risk factor for renal function loss^22^. The activation of RAGE leads to a sequence of signaling with activation of inflammatory response^1^. 

Renal tissue (±150 mg) was homogenized (ULTRA-TURRAX^®^ T 25 basic IKA^®^ Werke, Staufen, Germany) in 1.0 mL of phosphate-buﬀered saline (PBS) pH 7.4 cold solution and centrifuged at 800 g at 4°C for 10min. The supernatant (100 µL) was used in the analysis. Tumoral necrosis factor-alpha (TNF-α), interleukin-6 (IL-6), and monocyte chemoattractant protein-1 (MCP-1) levels were measured by ELISA method using commercial kits from R&D Systems, Minneapolis, USA (TNF-α: DY 510; IL-6: DY 506; MCP-1). The TNF-α limit of detection was 4000-62.5 pg/mL, IL-6 limit of detection was 8000-125 pg/mL, and the MCP-1 limit was 1000-15.6 pg/mL. The supernatant (100 µL) was used for analysis, and the results were corrected according to the protein amount.

### RENAL PROTEIN CARBONYLATION

Carbonylation is an irreversible protein modification induced by reactive oxygen species (ROS). It can be produced by oxidative cleavage of the backbone of the protein or by an attack by ROS radicals on some specific amino acids in the side chains such as lysine, arginine, proline, or threonine. Protein carbonyls are the most widely used markers to measure oxidative protein damage^23^. 

The supernatant described above was used for renal protein carbonylation analysis. Carbonylated proteins were measured by an unspecific method that uses DNPH (2,4-dinitrophenylhydrazine derivatizing agent) and photometric detection of any modified protein by carbonylation^24^. Carbonylated protein levels are expressed in nmol of DNPH/mg of protein.

### RENAL NOX-4 GENE EXPRESSION

Of the NOX isoforms, Nox4 is abundantly expressed in the kidney and is an important source of renal ROS triggered by RAGE^25^. Total RNA was extracted from renal tissue using the reagent TRIzol (Invitrogen). The SuperScript II First-Strand Synthesis System for RT-PCR (Invitrogen) kit was utilized for the synthesis of 20 mL of complementary DNA from 1000 ng of total RNA. The mRNA levels of NOX-4 (assay Rn 00585380_m1; Applied Biosystems) were determined by real-time PCR. Quantitative measurements were made with a commercial kit (TaqMan qPCR; Applied Biosystems) in a detection system (StepOne Plus; Applied Biosystems). Cycling conditions were as follows: enzyme activation at 50 ºC for 2 min, denaturation at 95 ºC for 10 min; complementary DNA products were amplified for forty cycles of denaturation at 95 ºC for 15 s and annealing/extension at 60 ºC for 1 min. Gene expression was quantified in relation to the values of the Control group after normalization by an internal control (cyclophilin: assay Rn 00690933_m1; Applied Biosystems) by the method 22DDCT, as described previously^26^.

### RENAL FUNCTION

After the collection of 24-h urine from the metabolic cages, the renal function was evaluated considering the glomerular filtration rate (GFR = (urine creatinine × flux)/plasma creatinine)^10^ and the protein/creatinine ratio, since it reflects proteinuria and is considered a marker of kidney function^27^.

### STATISTICAL ANALYSIS

Results are reported as means ± standard deviation (SD) or median (interquartile range). Differences among the groups were determined by one-way analysis of variance. Statistically significant variables were subjected to the Tukey post-hoc test to compare all the groups. Statistical analyses were performed using Sigma Stat for Windows Version 3.5 (Systat Software Inc., San Jose, CA, USA). A p value of 0.05 was considered statistically significant.

## RESULTS

**Figure 1:**
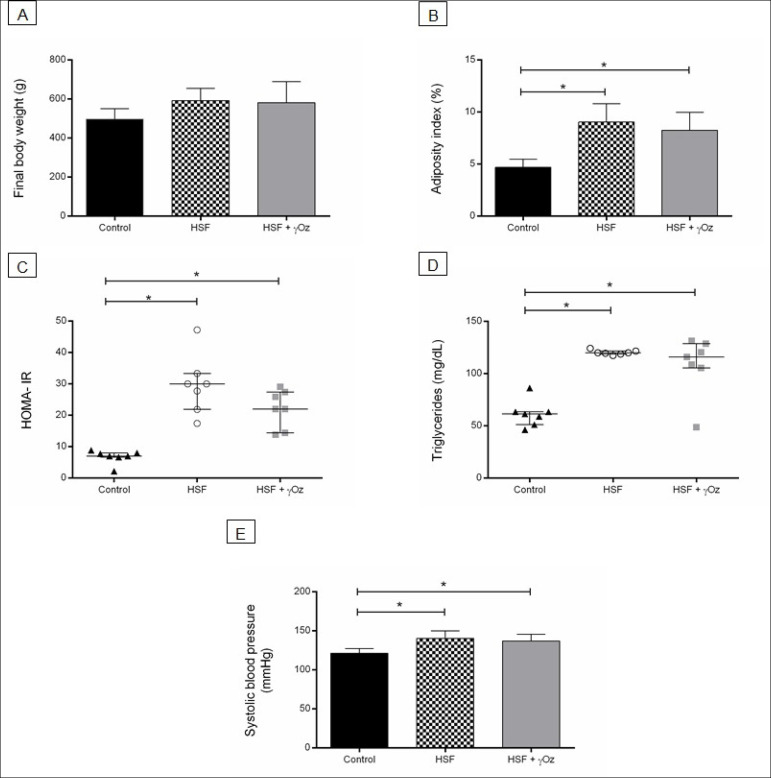
Nutritional and obesity-related disorders parameters at the end of 30 weeks. (A) Final body weight (g); (B) Adiposity index (%); (C) HOMAIR; (D) Plasma triglycerides levels (mg/dL); (E) Systolic blood pressure (mmHg). Data are expressed in means ± standard deviations or medians and interquartile ranges (n = 7 animals/group). Comparison by one-way ANOVA with Tukey post-hoc. *p < 0.05. HSF: high sugar-fat diet; HSF + γOz: high sugar-fat diet + gamma-oryzanol.

Figure 1 presents the nutritional parameters of groups. It is possible to verify that both groups that received HSF diet (HSF and HSF + γOz groups) presented increased adiposity index, insulin resistance, dyslipidemia, and systolic blood pressure compared to control group. No effect of γOz on these parameters was observed.

**Figure 2:**
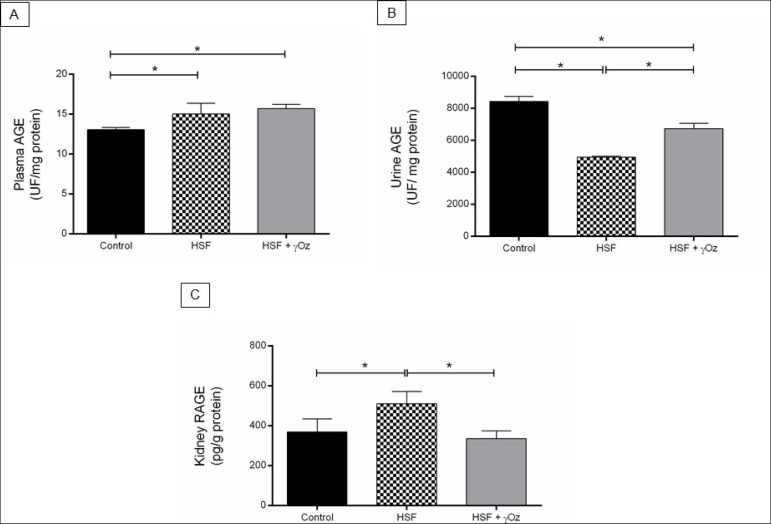
AGEs and RAGE levels. (A) Plasma AGEs levels (UF/mg protein); (B) Urine AGEs levels (UF/mg protein); (C) Kidney RAGE levels (pg/g protein) (C). Data are reported in means ± standard deviations (n = 7 animals/group). Comparison by one-way ANOVA with Tukey post-hoc test. *p < 0.05. HSF: high sugar-fat diet; HSF + γOz: high sugar-fat diet + gamma- oryzanol.

The plasma AGEs levels were the same in the HSF and HSF + γOz groups and higher than the control group. The HSF group presented lower urine AGE and increased kidney RAGE compared to the control group. The HSF + γOz group presented increased AGEs levels in urine and reduced RAGE levels compared to the HSF group (Figure 2).

**Figure 3:**
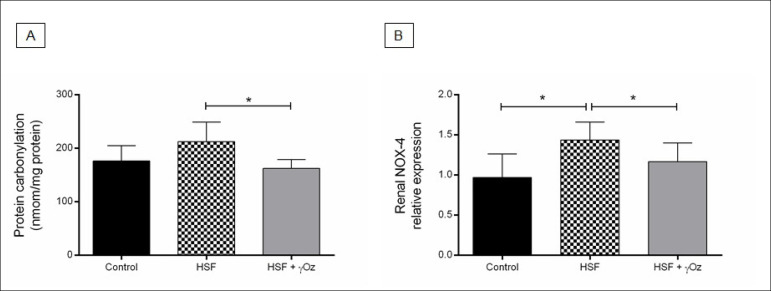
Kidney oxidative stress parameters. (A) Kidney protein carbonylation (nmol/mg protein); (B) NOX-4 relative gene expression. Data are reported in means ± standard deviations (n = 7 animals/group). Comparison by one-way ANOVA with Tukey post-hoc test. *p < 0.05. HSF: high sugar-fat diet; HSF + γOz: high sugar-fat diet + gamma-oryzanol.

Kidney oxidative stress parameters are presented in Figure 3. The HSF group presented increased NOX-4 gene expression compared to the control group. The treatment with gamma-oryzanol was effective to reduce carbonylation and NOX-4 gene expression in the HSF + γOz compared to the HSF group.

**Figure 4:**
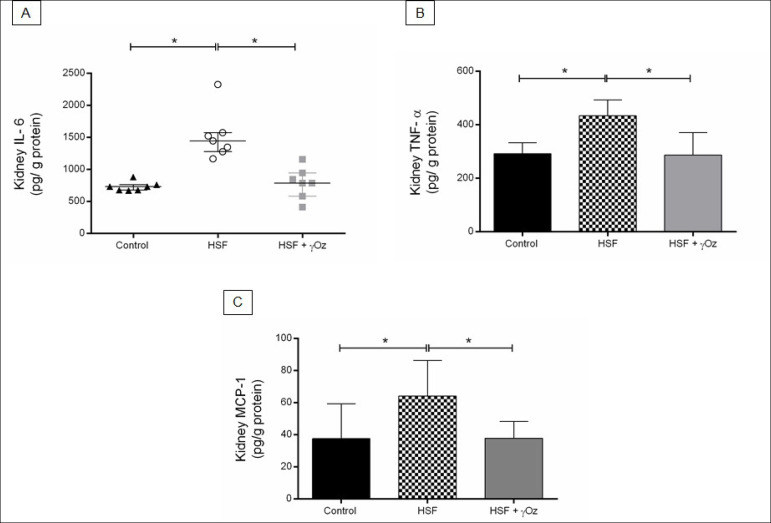
shows the renal inflammatory parameters. The HSF group presented increased pro-inflammatory parameters levels in comparison to the control group while the HSF + γOz group presented lower IL-6, TNF-α, and MCP-1 levels than the HSF group.

Figure 4. Kidney inflammatory parameters. (A) Interleukin-6 (IL-6, pg/g protein); (B) Tumoral necrosis factor alpha (TNF-α, pg/g protein); (C) Monocyte chemoattractant protein -1 (MCP-1, pg/g protein). Data are reported in means ± standard deviations or medians and interquartile ranges (n = 7 animals/group). Comparison by one-way ANOVA with Tukey post-hoc test. *p < 0.05. HSF- high sugar-fat diet; HSF + γOz- high sugar-fat diet + gamma-oryzanol.

**Figure 5:**
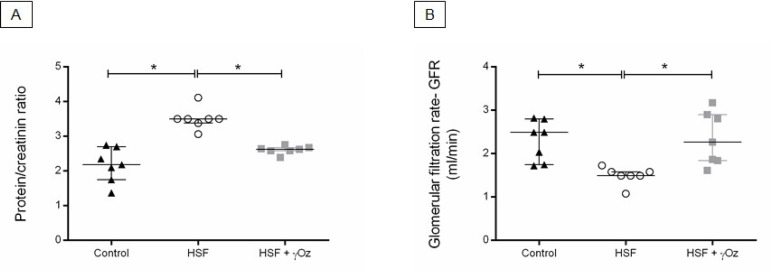
Renal function parameters. (A) Urine protein/creatinine ratio; (B) Glomerular filtration rate (GFR, mL/min). Data are reported in medians and interquartile ranges (n = 7 animals/group). Comparison by one-way ANOVA with Tukey post-hoc test. *p < 0.05. HSF: high sugar-fat diet; HSF + γOz: high sugar-fat diet + gamma-oryzanol.

The renal function parameters are presented in Figure 5. The HSF group presented increased protein/creatinine ratio and lower GFR compared to the control group. Otherwise, the HSF + γOz group presented improvement in kidney function, characterized by increased glomerular filtration rate. This group also presented reduction in kidney injury, with lower proteinuria (protein/ creatinine ratio) compared to the HSF.

## DISCUSSION

The aim of this study was to evaluate the effect of γOz in reducing renal inflammation and oxidative stress by modulating the AGEs/RAGE axis in animals submitted to a high sugar-fat diet. Obesity is a condition associated with several disorders considered risk factors for renal disease development, among them, central obesity, increased triglyceride levels, low high-density lipoproteins, hypertension, and elevated fasting glucose^28^. Some possible pathways for inducing kidney disease are insulin resistance and chronic inflammation, a major contributor to microvascular remodeling; dyslipidemia and excessive nutrient availability that may induce mitochondrial dysfunction; adipokines unbalance; the renin-angiotensin system; and oxidative stress. Our results showed no effect of γOz on the metabolic parameters, in contrast to the literature which shows positive effects, especially on glucose levels and dyslipidemia. This divergence can be explained by the use of different animal models, dose of γOz, and time of treatment. Our study treated male Wistar rats with 0.5% γOz in the chow for 10 weeks, a dose correspondent to an average consumption of 50 mg/day of γOz. Wang et al. (2015)^29^. fed male Sprague Dawley rats with a high-fat and high-fructose diet supplemented with 0.05% of FA or 0.16% of γOz for 13 weeks and they found that FA and γOz exhibited similar effects in alleviating obesity, hyperlipidemia, hyperglycemia, and insulin resistance. Cheng et al. (2013)^30^ studied the effect of a diet with 15% of palm oil with the addition of 5.25 g of gamma-oryzanol for 5 weeks in male Wistar rats with type 2 diabetes induced by streptozotocin. No effect of γOz was observed on body fat, glucose, and insulin; however, insulin resistance (area under the curve), triglycerides, and LDL cholesterol levels were reduced with the compound. 

The role of inflammation in chronic kidney disease (CKD) pathogenesis and progression has been recognized since the late 1990s when IL-1 levels were associated with major complications and increased rate of mortality in patients undergoing chronic dialysis. Following studies have demonstrated that persistent inflammation is able to promote adverse consequences to kidneys^31^. Evidence shows that inflammation and inflammatory reactions from any cause can modify or interfere with the intrarenal microcirculatory regulation and perfusion distribution and can induce renal damage, thus enhancing kidney disease progression^31^. Our results showed that the inflammatory response in the kidney was attenuated in the HSF group treated with γOz , corroborating the anti-inflammatory effect of the compound.

Oxidative stress pathogenesis in CKD patients has been well documented in the current literature^32^. Excessive production of ROS results in the activation of several enzymatic systems such as nicotinamide adenine dinucleotide phosphate (NADPH) oxidase and the mitochondrial respiratory chain, and, together with impaired antioxidant defense mechanisms, are the main factors for the oxidative stress condition that occurs in CKD, which leads to oxidation of macromolecules, tissue damage, and dysfunction. Therefore, excess generation of ROS has been directly linked to disease mechanisms and processes associated with CKD initiation and progression, including proteinuria, arterial hypertension, and diabetes mellitus. Moreover, the association among oxidative stress and chronic and endothelial dysfunction maintain and perpetuate the vicious circle where chronic kidney damage generates more kidney injury and systemic complications of CKD, as cardiovascular dysfunction. Evidence shows that oxidative stress is already present even in the early stages of CKD with increased NADPH oxidase production, especially NADPH subunit NOX-4. Oxidized lipoprotein particles, as carbonylated proteins, have been shown to accumulate in CKD as renal dysfunction progresses 33. Thus, since our results show that the HSF + γOz group presented reduced protein carbonylated and NOX-4 levels compared to the HSF group, we can confirm the antioxidant effect of γOz.

The metabolic changes present in the HSF groups are able to lead to renal inflammation and oxidative stress activation pathways. However, even with no effect of γOz, the treated animals showed reduced inflammation and oxidative stress. This can be explained by the effect of γOz on modulating the AGEs/RAGE axis in the HSF + γOz animals. AGEs are complex fluorescent products and they are formed through different pathways involving a direct glycation followed by re-arrangements or by the reaction of reactive carbonyl species (RCS) such as glyoxal, methylglyoxal, and 3-deoxyglucosone, which are formed through degradation pathways of sugars. Based on the AGEs formation mechanism, we can consider that in the present animal model, AGEs are formed by the increased sugars present in the diet leading to a direct protein glycation and then fluorescent products by re-arrangement reactions. AGEs induce oxidative stress through RAGE activation as demonstrated here by NOX-4 upregulation, which further sustains AGEs formation by forming RCS species through reducing sugar oxidation. Oxidative stress also promotes protein carbonylation and advanced lipoxidation end products (ALEs) formation, which result either from a direct oxidation or by RCS from lipid peroxidation, such as 4- hydroxynonenal (HNE), MDA, and acrolein^34^. The above-mentioned events were confirmed in the study, and in particular we found that the increase of RAGE and NOX-4 expression in the kidney were accompanied by an increase of protein carbonylation.

AGEs were found to be reduced in the urine of HSF in comparison to control animals, and this can be explained by considering a reduced excretion of these protein adducts. The mechanism explaining this reduction is being evaluated, and one explanation could be that the AGEs are trapped by RAGE whose content has increased in the kidney of HSF animals, thus reducing the urinary content of the AGEs. Hence in HSF animals the AGEs/RAGE axis is activated leading to an oxidative stress response, which promotes an inflammatory condition as observed by finding an increase of IL-6, TNF-α, and MCP-1. γOz was found to significantly reduce all the above-mentioned events and in particular the AGEs/RAGE axis by increasing the urinary excretion of AGEs and reducing RAGE expression, and the oxidative damage, by reducing protein oxidation and finally the anti-inflammatory response^35^. The present paper does not indicate through which mechanisms γOz elicits such a protective mechanism but can be speculated based on the literature. Very recently, Sobhy et al. (2020)^15^ found that oryzanols inhibit glycation and AGEs formation by a direct anti-glycation effect and by scavenging the free radicals generated during the glycation reactions. Furthermore, the antioxidant activity of γOz has been reported in both in vitro and in vivo experiments by several groups^10^^,^ 36 ^,^ 37.

In summary, this study found that the group treated with gamma-oryzanol showed reduced kidney RAGE levels, inflammation, and oxidative stress and increased renal AGEs excretion probably due the positive effect on glomerular filtration rate. Thus, it is possible to conclude that the gamma-oryzanol was effective in reducing inflammation and oxidative stress in the kidney by modulating the AGEs/RAGE axis.

It is important to report some limitations of this study. Only general, and not specific AGEs or RAGE, were analyzed, more oxidative stress and inflammation markers could be included in the parameters analyzed, the immunohistochemical analysis for RAGE could have been performed, as well as histological analysis of the kidneys to demonstrate possible damage to structures. 
